# Estimation of cancer burden in Guangdong Province, China in 2009

**DOI:** 10.1186/s40880-015-0060-4

**Published:** 2015-11-16

**Authors:** Su-Mei Cao, Yan-Jun Xu, Guo-Zhen Lin, Qi-Hong Huang, Kuang-Rong Wei, Shang-Hang Xie, Qing Liu

**Affiliations:** State Key Laboratory of Oncology in South China, Collaborative Innovation Center for Cancer Medicine, Sun Yat-sen University Cancer Center, Guangzhou, 510060 Guangdong P.R. China; Department of Cancer Prevention, Sun Yat-sen University Cancer Center, Guangzhou, 510060 Guangdong P.R. China; Institute of Control and Prevention for Chronic Non-infective Disease, Guangdong Provincial Center for Disease Control and Prevention, Guangzhou, 511430 Guangdong P.R. China; Department of Disease Surveillance, Guangzhou Center for Disease Control and Prevention, Guangzhou, 510440 Guangdong P.R. China; Sihui Institute of Cancer, Sihui, 530000 Guangdong P.R. China; Institute of Cancer, Zhongshan People’s Hospital, Zhongshan, 528403 Guangdong P.R. China

**Keywords:** Disease burden, Incidence-to-mortality ratio, Log-linear model

## Abstract

**Background:**

Surveying regional cancer incidence and mortality provides significant data that can assist in making health policy for local areas; however, the province- and region-based cancer burden in China is seldom reported. In this study, we estimated cancer incidence and mortality in Guangdong Province, China and presented basic information for making policies related to health resource allocation and disease control.

**Methods:**

A log-linear model was used to calculate the sex-, age-, and registry-specific ratios of incidence to mortality (I/M) based on cancer registry data from Guangzhou, Zhongshan, and Sihui between 2004 and 2008. The cancer incidences in 2009 were then estimated according to representative I/M ratios and the mortality records from eight death surveillance sites in Guangdong Province. The cancer incidences in each city were estimated by the corresponding sex- and age-specific incidences from cancer registries or death surveillance sites in each area. Finally, the total and region-based cancer incidences and mortalities for the entire population of Guangdong Province were summarized.

**Results:**

The estimated I/M ratios in Guangzhou (3.658), Zhongshan (2.153), and Sihui (1.527) were significantly different (*P* < 0.001), with an average I/M ratio of 2.446. Significant differences in the estimated I/M ratios were observed between distinct age groups and the three cancer registries. The estimated I/M ratio in females was significantly higher than that in males (2.864 vs. 2.027, *P* < 0.001). It was estimated that there were 163,376 new cancer cases (99,689 males and 63,687 females) in 2009; it was further estimated that 115,049 people (75,054 males and 39,995 females) died from cancer in Guangdong Province in 2009. The estimated crude and age-standardized rate of incidences (ASRI) in Guangdong Province were 231.34 and 246.87 per 100,000 males, respectively, and 156.98 and 163.57 per 100,000 females, respectively. The estimated crude and age-standardized rate of mortalities (ASRM) in Guangdong Province were 174.17 and 187.46 per 100,000 males, respectively, and 98.59 and 102.00 per 100,000 females, respectively. In comparison with the western area and the northern mountain area, higher ASRI and ASRM were recorded in the Pearl River Delta area and the eastern area in both males and females.

**Conclusions:**

Cancer imposes a heavy disease burden, and cancer patterns are unevenly distributed throughout Guangdong Province. More health resources should be allocated to cancer control, especially in the western and northern mountain areas.

## Background

Cancer is the leading cause of death in China [1–4]. Analyzing the cancer burden in a population can provide valuable information for collecting etiological data and developing cancer control policies. In China, social and economic changes and an aging population have contributed to rapid increases in the morbidity and mortality of most cancers. It has been observed that different provinces/areas in China have uneven trends in cancer development because of special risk factors [5, 6]. Thus, surveying regional cancer incidence and mortality trends can provide valuable data to assist in making health policy for local areas. The National Central Cancer Registry has published Chinese national cancer incidence and mortality statistics since 2008 [7]; however, the province- and region-based cancer burden is rarely reported.

Guangdong Province has a highly developed economy and a population of approximately 100 million; however, the level of economic development varies throughout the province. The Pearl River Delta area (including Guangzhou, Shenzhen, Zhuhai, Foshan, Zhongshan, Dongguan, Huizhou, Zhaoqing, and Jiangmen), which is a floodplain in the center of Guangdong Province, and the eastern area (including Shantou, Chaozhou, Jieyang, and Shanwei) of Guangdong Province are economically well developed (i.e., average income), industrialized, populous, and polluted. In comparison, in the northern mountain area (including Shaoguan, Qingyuan, Meizhou, Heyuan, and Yunfu) and the western area (including Zhanjiang, Maoming, and Yangjiang) of the province, the level of economic development is lower, and the population is sparse, but the environmental pollution is lower. Differences in population, economy, industrialization, and lifestyle in various areas of Guangdong Province may influence the risk of cancer.

No provincial cancer incidence data exist for Guangdong Province up to now, which limits the evidence available for making cancer control policies. Since 1997, four cancer registry sites have been established in Guangdong Province. These sites cover a population of approximately 20 million, including two big cities (Guangzhou and Shenzhen) and two medium-sized cities (Zhongshan and Sihui); however, these cities are concentrated in the Pearl River Delta area. The uneven distribution of cancer registry sites means that the cancer registry data cannot depict a representative picture of the provincial cancer burden. Recently, three new cancer registry sites were built in Guangdong Province, but they lack precision and completeness with the cancer incidence too low or the incidence-to-mortality (I/M) ratio extremely high. Fortunately, eight death surveillance sites in Guangdong Province are distributed in more areas, including the northern mountain, western, and eastern areas of the province. Estimating the incidence and mortality of cancer using combined data from the cancer registries and death surveillance sites in Guangdong Province will provide more accurate data.

Jensen et al. [8] developed a statistical technique for estimating the incidence of cancer using mortality data. They used a model of mortality as a Poisson regression with the log incidence as an offset to estimate the incidence of cancer based on the number of cancer deaths. This model has been applied comprehensively to estimate the cancer burden in European and other countries [2, 9, 10]. Chen et al. [11] used a generalization of this method to estimate the cancer burden in China. They fitted generalized linear mixed models and estimated the parameters of models by a Bayesian Markov chain Monte Carlo method. In the present study, we estimated the total and regional cancer burden in Guangdong Province in 2009 using aggregate data from the cancer registry and death surveillance sites.

## Methods

### Data source

#### Incidence and mortality

Since 1997, four cancer registry sites have existed in Guangzhou, Shenzhen, Zhongshan, and Sihui in Guangdong Province. We collected incidence and mortality data between 2004 and 2008 from each registry site. After evaluating the quality of the cancer registries according to the standards defined by the International Agency for Research on Cancer [12], we determined that the completeness and reliability of mortality data from Shenzhen did not meet the standards; therefore, the data from Shenzhen were excluded from the development of an estimation model for the I/M ratio. Thus, data from three cancer registries were included in the estimation model. The reported cancer spectrum in the cancer registries contained all types of cancer (ICD-10 C00-C90).

Cancer mortality data were collected from the Guangdong Provincial Center of Disease Control and Prevention. The data were from eight death surveillance sites in Guangdong Province, located in the Yuexiu district of Guangzhou, Zhuhai, Taishan, Nanxiong, Yunfu, Shanwei, Sihui, and Wuhua. The mortality data from Yuexiu of Guangzhou and Sihui overlapped the data from the cancer registries. Therefore, only one set of data was included in the analysis (Table 1).

**Table 1:** The economic partitions and covered areas of the cancer registry and death surveillance data sources in Guangdong Province, China

Economic area	City	Cancer registry	Death surveillance site
Pearl River Delta	Guangzhou	Guangzhou	
	Shenzhen	Shenzhen	
	Zhuhai		Zhuhai
	Foshan	Zhongshan	
	Zhongshan	Zhongshan	
	Dongguan	Zhongshan	
	Huizhou	Zhongshan	
	Zhaoqing	Sihui	
	Jiangmen		Taishan
Eastern	Shantou		Shanwei
	Chaozhou		Shanwei
	Jieyang		Shanwei
	Shanwei		Shanwei
Western	Zhanjiang		Yunfu
	Maoming		Yunfu
	Yangjiang		Taishan
Northern mountain	Shaoguan		Nanxiong
	Qingyuan		Nanxiong
	Meizhou		Nanxiong
	Heyuan		Nanxiong
	Yunfu		Yunfu

#### Population data

Population and age distribution data were obtained from the Guangdong statistics annual report, published by the Guangdong Statistics Bureau [13]. The estimated provincial population in 2009 was 83,659,800, including 43,091,000 males and 40,568,800 females. Regional population data in 2009 were extracted by sex and age (grouped into 5-year age ranges, e.g., 0–4, 5–9, 10–14, … 80–84, and 85+ years). The estimated populations were 29,670,200 in the Pearl River Delta area, 17,583,400 in the eastern area, 17,741,200 in the western area, and 18,665,000 in the northern mountain area [13].

### Statistical methods

Since the I/M ratio is a fairly stable value in a large, defined population, it is used as an index to evaluate the quality of cancer registry data. Furthermore, it can be used to estimate cancer incidence and mortality for the same population [10, 11]. In our study, we estimated cancer incidence in different parts of Guangdong Province using the combination of mortality and the I/M ratio. The I/M ratios were estimated from log-linear models of the number of incident cases based on the aggregation of cancer registries in Guangzhou, Zhongshan, and Sihui between 2004 and 2008, offset by the number of deaths in the same registries and adjusted for sex and age. The regional incidence number (IN) in 2009 were obtained by applying a set of sex-, age-, city-specific I/M ratios and the estimated regional mortality number (MN) as follows: IN = MN × (I/M). Before aggregation, each registry was weighted to account for the relative size of the population.

A log-linear regression model was developed for each sex. The age groups were indexed by i (i = 1, …, 18), and the registries were indexed by j (j = 1, 2, 3). The I/M ratio was expressed as the $\log ({\text{I/M}}) = \mu_{j} + \beta_{i}$, where *β*_i_ represents the log of the mean age-specific I/M ratio, and *μ*_j_ represents the random intercept for different registries. Then, the regional incidence was estimated by using the formula exp (*μ*_j_ + *β*_i_) × mortality in each age group in each area.

For each city in Guangdong Province that was studied, the number of cancer cases and deaths in 2009 were estimated by multiplying the predicted incidence and mortality by the corresponding city populations. The cancer registry and death surveillance sources for the estimated incidences and mortalities in the cities in Guangdong are listed in Table 1. The rates were calculated by using the age-standardized rate per 100,000, based on the standard population of China in 2000.

We were authorized to use the data from the cancer registry and death surveillance sites for this analysis. No personal information, such as name, identification number, home address, and personal contact details, was included when the analyzed data were extracted from the database. Given the highly aggregated form of data extraction, ethics approval was not required for this study.

## Results

### The I/M ratios for cancers in Guangdong Province

**Fig. 1:**
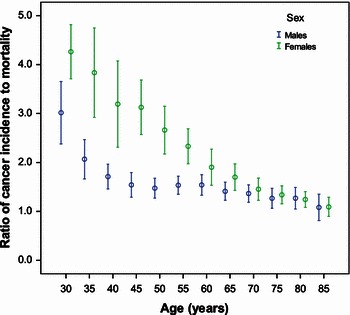
The estimated age-specific ratios of cancer incidence to mortality in males and females in three cities (Guangzhou, Zhongshan, and Sihui) using a log-linear regression model in Guangdong Province, China. °, mean of ratio; , 95% confidence interval

Based on the cancer incidence and mortality data from the three cancer registries (Guangzhou, Zhongshan, and Sihui) between 2004 and 2008, the estimated average I/M ratio was 2.446 [95% confidence interval (CI) 2.324–2.568]. The sex- and registry site-adjusted I/M ratios are shown in Table 2. Males had a lower I/M ratio than females (2.027, 95% CI 1.856–2.199 in males and 2.864, 95% CI 2.691–3.038 in females). The I/M ratio in Guangzhou (3.658, 95% CI 3.457–3.859) was the highest; the I/M ratio in Sihui (1.527, 95% CI 1.294–1.759) was the lowest. The age-specific I/M ratio showed an overall declining trend in males and females (Fig. 1); however, the age-specific I/M ratio in males reached a plateau after the 55- to 60-year age range and approached the level of I/M ratio in females.

**Table 2:** The I/M ratios from the three cancer registries in Guangzhou, Zhongshan, and Sihui in Guangdong Province, China in 2004–2008

Variable	Mean	Standard deviation	95% confidence interval
Sex			
Male	2.027	0.087	1.856–2.199
Female	2.864	0.088	2.691–3.038
City			
Guangzhou	3.658	0.102	3.457–3.859
Zhongshan	2.153	0.101	1.953–2.352
Sihui	1.527	0.118	1.294–1.759
Average	2.446	0.062	2.324–2.568

Table 3 shows the I/M ratios of cancers in the three cities in 2009. A log-linear regression model was used to estimate the corresponding cancer incidences in six cities (Zhuhai, Taishan, Nanxiong, Wuhua, Shanwei, and Yunfu) by using the local death surveillance data and the I/M ratios from Sihui or Zhongshan (Table 4).

**Table 3:** The I/M ratio in Guangzhou, Zhongshan, and Sihui, China in 2009 (per 100,000)

City	Males					Females				
	CI	ARSI	CM	ASRM	I/M	CR	ASRI	CM	ASRM	I/M
Guangzhou	355.86	273.87	252.81	188.30	1.41	301.64	213.48	155.61	98.38	1.94
Zhongshan	243.47	207.74	176.01	150.22	1.38	156.78	122.47	80.43	61.19	1.95
Sihui	266.38	284.30	189.26	203.18	1.41	190.09	164.65	99.50	82.37	1.91

*CI* crude incidence, *ASRI* age-standardized rate of incidence, *CM* crude mortality, *ASRM* age-standardized rate of mortality

**Table 4:** The mortalities and estimated incidences of cancers in Zhuhai, Taishan, Nanxiong, Wuhua, Shanwei, and Yunfu in Guangdong Province, China in 2009 (per 100,000)

City	Males				Females			
	CR	ASRI	CM	ASRM	CR	ASRI	CM	ASRM
Zhuhai^a^	129.88	138.54	98.65	104.49	123.12	128.77	66.15	68.42
Taishan^a^	200.34	160.82	151.87	116.04	127.77	109.92	66.81	52.09
Nanxiong^b^	180.63	182.25	147.96	148.80	118.20	102.62	78.43	63.39
Wuhua^b^	162.54	178.94	134.80	146.42	89.62	94.70	61.71	62.32
Shanwei^a^	194.89	270.15	152.64	209.99	117.65	158.80	74.02	91.38
Yunfu^b^	159.23	189.31	133.60	158.73	109.95	103.69	77.09	67.77

Abbreviations as in Table 3

^a^The incidence was estimated using the I/M ratio of Zhongshan

^b^The incidence was estimated using with the I/M ratio of Sihui

### Estimated cancer incidence and mortality in Guangdong Province

Cancer incidence data from three cancer registries and cancer mortality data from six death surveillance sites were used to estimate the cancer incidences of the 21 cities in Guangdong Province and then to aggregate the cancer burden in the four economic areas and in the entire province. The cancer incidences in the four areas and the entire Guangdong Province in 2009 are shown in Table 5. The estimated total new cases of cancer in 2009 was 163,376, including 60,352 in the Pearl River Delta area, 39,796 in the eastern area, 30,679 in the western area, and 32,550 in the northern mountain area. The estimated crude incidence and age-standardized rate of incidence (ASRI) of cancer were 231.34 and 246.87 per 100,000 males, respectively, and 156.98 and 163.57 per 100,000 females, respectively. In the four economic areas of Guangdong Province, the ASRI in males was the highest in the eastern area (307.29 per 100,000), followed by the Pearl River Delta area (269.04 per 100,000) and the western area (212.51 per 100,000); it was the lowest in the northern mountain area (206.19 per 100,000). In comparison, the highest ASRI of cancer in females was 208.81 per 100,000 in the Pearl River Delta area, followed by 192.26 per 100,000 in the eastern area, 120.39 per 100,000 in the western area, and 115.25 per 100,000 in the northern mountain area.

**Table 5:** The estimated incidences of cancers in different economic areas of Guangdong Province, China in 2009 (per 100,000)

Age	Pearl River Delta area		Eastern area		Western area		Northern mountain area		Total	
	Males	Females	Males	Females	Males	Females	Males	Females	Males	Females
0–	13.12	11.42	0.00	0.00	1.52	0.00	22.33	16.33	9.89	7.52
5–	11.10	4.83	14.44	0.00	3.00	0.00	6.07	0.00	8.45	1.29
10–	9.31	8.87	4.99	18.03	1.13	1.28	11.44	4.73	6.29	8.83
15–	16.11	14.17	43.48	0.00	12.99	3.32	12.15	13.07	21.32	7.85
20–	20.95	20.98	20.21	0.00	18.47	3.49	16.77	5.03	19.68	11.15
25–	31.55	33.29	0.00	25.20	13.06	63.40	20.22	28.24	20.47	35.80
30–	43.70	59.68	34.92	22.64	77.84	28.93	45.27	39.51	48.42	43.69
35–	100.40	121.47	144.97	194.75	108.40	66.72	127.02	66.20	115.16	113.35
40–	199.18	215.82	148.53	88.37	112.35	211.18	187.21	102.58	171.00	164.52
45–	261.48	285.85	310.29	88.25	245.70	120.84	282.85	195.39	273.03	191.04
50–	495.54	430.46	549.58	618.31	355.78	220.15	324.67	191.34	433.34	366.45
55–	844.85	609.30	970.50	793.85	534.70	371.16	577.29	332.40	734.95	534.29
60–	909.10	551.38	1332.26	318.87	661.70	124.18	690.18	279.68	880.88	347.81
65–	1126.07	610.51	1290.79	500.86	603.24	241.37	756.13	347.44	916.73	430.15
70–	1440.00	879.27	1726.85	611.40	1466.52	405.04	1278.88	471.72	1457.02	601.42
75–	1621.16	899.10	1847.73	1274.01	1719.95	914.12	970.90	577.31	1513.55	882.38
80–	1785.34	981.55	1498.50	1085.43	1829.40	946.54	899.74	495.86	1514.33	858.71
85–	1643.92	730.28	673.23	491.75	661.96	525.41	414.23	392.00	852.96	541.65
Total	221.16	185.01	276.29	174.36	215.33	125.00	221.12	124.79	231.34	156.98
ASRI	269.04	208.81	307.29	192.26	212.51	120.39	206.19	115.25	246.87	163.57

*ASRI* age-standardized rate of incidence

Table 6 shows the estimated crude mortality and age-standardized rate of mortality (ASRM) of cancer in the four economic areas and in the entire Guangdong Province in 2009, including 37,539 in the Pearl River Delta area, 28,566 in the eastern area, 23,857 in the western area, and 25,088 in the northern mountain area. It was estimated that 115,049 people died from cancer in Guangdong in 2009. The crude mortality and ASRM were 174.17 and 187.46 per 100,000 males, respectively, and 98.59 and 102.00 per 100,000 females, respectively. Just like the distribution of cancer incidence, the ASRM of cancer in males was the highest in the eastern area of the province (238.69 per 100,000), but the ASRM of cancer in the Pearl River Delta area was close to that in other areas, with 177.98 per 100,000 in the delta area, 170.55 per 100,000 in the western area, and 168.91 per 100,000 in the northern mountain area. The highest ASRM of cancer in females was 126.78 per 100,000 in the Pearl River Delta area, followed by 113.75 per 100,000 in the eastern area, 79.36 per 100,000 in the western area, and 75.15 per 100,000 in the northern mountain area.

**Table 6:** The estimated mortalities of cancers in different economic areas of Guangdong Province, China in 2009 (per 100,000)

Age	Pearl River Delta area		Eastern area		Western area		Northern mountain area		Total	
	Males	Females	Males	Females	Males	Females	Males	Females	Males	Females
0–	5.21	6.80	0.00	0.00	0.75	0.00	16.74	8.99	6.09	4.29
5–	5.67	3.63	8.40	0.00	1.75	0.00	4.82	0.99	5.03	1.21
10–	8.45	7.80	4.60	5.41	1.04	0.39	9.08	3.55	5.45	4.32
15–	5.82	6.25	19.87	0.00	7.77	0.70	10.98	7.38	10.90	3.62
20–	6.19	8.79	8.30	0.00	9.31	1.41	10.80	3.46	7.91	4.88
25–	9.26	15.72	0.00	5.80	5.66	21.33	11.20	11.94	7.22	14.02
30–	20.81	29.97	18.36	5.97	47.44	9.85	31.68	15.76	27.06	19.35
35–	47.01	52.94	75.15	59.79	63.78	28.77	88.88	29.71	63.51	44.92
40–	97.10	90.77	92.67	28.40	75.20	98.83	142.90	67.68	103.05	75.23
45–	162.91	157.63	230.28	30.77	184.65	54.72	215.79	92.11	193.40	95.53
50–	317.51	250.32	414.08	298.40	284.21	136.06	278.01	96.07	321.12	197.83
55–	458.97	308.95	712.36	384.62	402.50	221.60	448.14	189.66	499.05	278.86
60–	570.33	347.89	1017.34	194.23	509.02	81.79	535.89	212.79	639.45	227.67
65–	829.67	436.28	1024.43	339.64	500.55	181.18	667.70	269.46	735.36	312.09
70–	1066.07	667.73	1459.35	490.20	1264.56	338.32	1125.39	418.52	1210.05	485.95
75–	1426.58	704.78	1787.77	1226.99	1664.11	882.32	939.36	482.03	1427.64	782.72
80–	1523.39	711.60	1568.15	1054.71	1727.52	861.73	794.34	438.30	1405.02	739.10
85–	1595.98	651.34	845.07	704.85	695.05	595.55	370.01	337.05	873.45	566.12
Total	142.57	109.88	215.93	106.86	177.17	86.23	181.98	83.92	174.17	98.59
ASRM	177.98	126.78	238.69	113.75	170.55	79.38	168.91	75.15	187.46	102.00

*ASRM* age-standardized rate of mortality

### The age distributions of cancer incidence and mortality

**Fig. 2:**
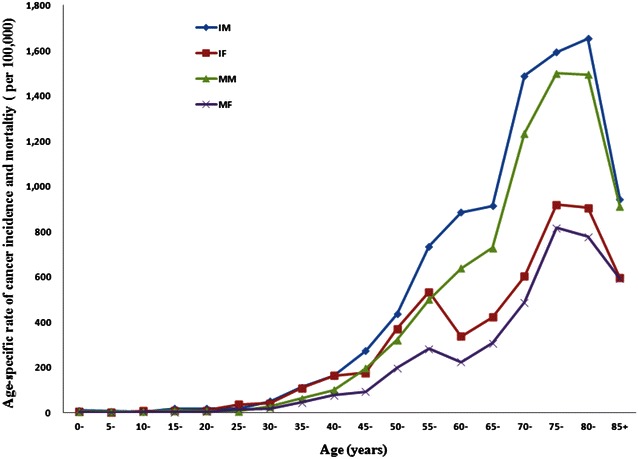
The estimated age-specific rates of cancer incidences and mortalities in males and females using aggregate data from the cancer registry and death surveillance sites in Guangdong Province, China in 2009. *IM* incidence in males, *IF* incidence in females, *MM* mortality in males, *MF* mortality in females

Figure 2 shows the age-specific rates of cancer incidence and mortality curves in Guangdong Province. In males, the age-specific rates of cancer incidence and mortality increased sharply after 30 years of age and reached a peak at 75–80 years of age. In females, the incidence and mortality had two peaks: one minor peaked around age 50–55 years and then declined in the subsequent 5–10 year group ranges; thereafter, cancer incidence and the mortality increased again until age 75–80 years.

## Discussion

In this study, we estimated the total I/M ratio of cancers in Guangdong Province in 2009 using a log-linear model based on the aggregation of cancer registry and death surveillance data. The population involved in the cancer registries and death surveillance sites was 12.86 million, encompassing 15.4% of the total population of Guangdong [13]. The death surveillance sites were distributed in all areas of Guangdong, which made the estimation more representative and precise. Owing to the difference in the I/M ratios among different sex and age groups, we also used sex- and age-specific parameters in the model. Therefore, the estimation reflects the realistic cancer burden in Guangdong Province and provides valuable information for developing cancer prevention and control policies.

It has been reported that the I/M ratios of cancers differ significantly in different registries, sexes, and age groups [9, 10]. Indeed, our results, based on data from three cancer registry sites, showed that the average I/M ratios in Guangzhou, Zhongshan, and Sihui were clearly distinguished. The I/M ratio in Guangzhou was the highest, followed by Zhongshan; Sihui had the lowest I/M ratio. The estimated I/M ratio was consistent with the regional economic development level and health resources. Guangzhou is the capital of Guangdong Province and congregated high-level general hospitals and tumor hospitals. High quality of health service may result in a higher average I/M ratio in Guangdong (2.466) than in the country as a whole (1.35) [9]. Therefore, the I/M ratio in Guangzhou is not suitable for other cities in Guangdong Province. We used the I/M model of Zhongshan to estimate the cancer incidence in medium-sized cities, such as Zhuhai, Taishan, and Shanwei; we used the I/M model of Sihui to estimate the cancer incidence in other cities.

With increasing age from 30 years until 85 years old, the I/M ratio of cancer decreased in both males and females. The trend in females was almost linear; however, there was a plateau in males at 40–60 years of age. The high incidences and mortalities of liver and lung cancer in this age range of males may explain the results. The results showed that the estimated crude cancer incidences in Guangdong Province were 231.34 per 100,000 males and 156.98 per 100,000 females in 2009, with 163,376 new cancer cases occurring annually (99,689 males and 63,687 females). These crude cancer incidences in Guangdong Province are higher than the nationwide average and close to the incidences in urban areas throughout the entire country [9].

According to the areal relationship and economic developing level, Guangdong Province was divided into four economic areas, including the eastern area, the Pearl River Delta area, the western area, and the northern mountain area. We analyzed the cancer incidences and mortalities in these areas. Higher cancer incidence and mortality in males and females were observed in the eastern area and the Pearl River Delta area; lower rates were observed in the western area and the northern mountain area. The higher cancer incidences and mortalities in the eastern area and the Pearl River Delta area may be related to a higher level of urbanization and industrialization, which brought more carcinogenetic contaminants to the environments.

From our results, the total numbers of cases and death in the eastern, western, and northern mountain areas accounted for more than 60% of all cancer cases in Guangdong in 2009. At present, cancer treatment resources are focused mainly on the Pearl River Delta area of Guangdong, especially Guangzhou and Shenzhen. This reflected that health resources distributed imbalance in Guangdong Province. We recommend that the allocation of medical resources and cancer treatment levels should be improved in the eastern, western, and northern mountain areas.

Our study showed that the estimated age-specific rates of cancer incidences and mortalities in Guangdong Province in 2009 increased monotonically with increasing age in males. However, in females, the age-specific rates of cancer incidences and mortalities had two peaks: one minor peaked around ages 50–55 years and then declined in the subsequent 5–10 year group ranges; thereafter, the cancer incidence and mortality increased again until ages 75–80 years. We suppose that this may be caused by an early exposure to common carcinogenic agents in females. For example, it has been reported that breast cancer, which is one of the most common cancers in Chinese women, has an earlier age of onset in Chinese women compared with women in Western countries [14]. This may be due to lifestyle differences in early life and differences of genetic background [15].

There are some limitations in this study. First, we only focused on the whole cancer burden in the dimension of cancer incidence and mortality and not to explore other dimension, such as cost of cancer treatment. Second, there is usually a 3-year delay in the publication of cancer registry data in China; therefore, we could use the data between 2004 and 2008 only to build the model of I/M ratio estimation and to estimate the incidence in 2009. In the future, more comprehensive and long-term cancer data should be collected, including cancer incidences and mortalities and survival data, so that more detailed and precise cancer burden analysis can be performed.
